# Knowledge and awareness of occupational therapy and associated factors among healthcare professionals in Rwanda: a cross-sectional study

**DOI:** 10.3389/fmed.2026.1722607

**Published:** 2026-10-20

**Authors:** Didier Ishimwe, Elysee Aubaine Nsengimana, Emmanuel Biracyaza, Pierre Damien Turikumana, Felix Niyonkuru, Potien Uwihoreye, Lizahn G. Cloete

**Affiliations:** 1 Department of Occupational Therapy, College of Medicine and Health Sciences, University of Rwanda, Kigali, Rwanda; 2 Rwanda Occupational Therapy Association (RWOTA), Kigali, Rwanda; 3 School of Rehabilitation, Faculty of Medicine, Université de Montréal, Montreal, QC, Canada; 4 Centre for Interdisciplinary Research in Rehabilitation of Greater Montreal (CRIR), Montreal, QC, Canada; 5 Department of Occupational Therapy, School of Rehabilitation Sciences, Boitekanelo College, Gaborone, Botswana; 6 Occupational Therapy Division, Department of Health and Rehabilitation Sciences, Faculty of Medicine and Health Sciences, Stellenbosch University, Cape Town, South Africa

**Keywords:** awareness, healthcare professional, hospital, knowledge, occupational therapy

## Abstract

**Background:**

Occupational therapy (OT) is a client-centered health profession that supports health and wellbeing by enabling individuals to engage in daily activities. In many low- and middle-income countries, including Rwanda, limited awareness and knowledge of OT among healthcare professionals (HCPs) hinder collaboration and access to services. Therefore, this study aimed to determine HCPs’ awareness and knowledge of OT and identify associated factors.

**Methods:**

A cross-sectional study was conducted among 201 HCPs from five teaching hospitals in Rwanda. Data were collected using a sociodemographic questionnaire and the Knowledge and Attitude Toward OT Practice Tool. Bivariate and multivariable logistic regression analyses were performed to determine factors associated with awareness and knowledge of OT. Associations were reported using adjusted odds ratios (aORs) with 95% confidence intervals (95% CIs), and statistical significance was set at *p* < 0.05.

**Results:**

Of the 201 HCPs, 102 (50.7%) were female, 74 (36.8%) were aged 31–40 years, and 40 (19.9%) were mental health nurses. Overall, 151 (75.1%) participants were aware of OT, while more than half (*n* = 104, 51.7%) exhibited poor knowledge. Physiotherapists [aOR = 6.3, 95% CI (1.6–25.5), *p* = 0.010], general nurses [aOR = 5.6, 95% CI (1.7–18.1), *p* = 0.004], and mental health nurses [aOR = 5.8, 95% CI (1.8–18.7), *p* = 0.003] reported higher likelihoods of awareness of OT than medical doctors. Awareness of OT was significantly associated with working in habilitation/rehabilitation services [aOR = 3.8, 95% CI (1.9–7.5), *p* < 0.001] and positive perceptions of OT. Similarly, good knowledge of OT was associated with profession, previous professional practice outside Rwanda, awareness of OT, and a positive perception of OT. Specifically, physiotherapists [aOR = 8.2, 95% CI (2.5–27.0), *p* = 0.001], ophthalmic clinical officers [aOR = 8.6, 95% CI (1.5–49.4), *p* = 0.016], general nurses [aOR = 3.2, 95% CI (1.2–8.6), *p* = 0.024], and mental health nurses [aOR = 2.7, 95% CI (1.1–7.3), *p* = 0.045] had higher odds of good knowledge of OT than medical doctors.

**Conclusion:**

Although awareness of OT was relatively high among HCPs, more than half demonstrated poor knowledge of the profession. Greater awareness and knowledge were associated with professional background, involvement in habilitation or rehabilitation services, previous professional practice outside Rwanda, direct experience working with occupational therapists, and positive perceptions of OT. Considering these results, our study highlights the need for strengthening interprofessional education and continuing professional development to improve understanding and integration of OT within the Rwandan healthcare system.

## Background

Occupational therapy (OT) promotes health and wellbeing by supporting participation in meaningful occupations that people want, need, or are expected to do (1). This profession enables individuals of all ages to engage fully in activities that give purpose and meaning to their lives, despite challenges related to physical, mental, or cognitive impairments. Its contribution is critical in diverse settings such as rehabilitation, disability management, mental health, pediatrics, geriatrics, and community-based care programs (2). Globally, the World Federation of Occupational Therapists (WFOT) recognizes OT as a cornerstone of inclusive and sustainable health and social systems. As of 2025, there are over 633,000 practicing occupational therapists worldwide, reflecting the rapid growth of this profession in response to complex global health needs (3). Despite more than a century of growth and development, OT continues to be misunderstood or underrecognized by the public and even among other healthcare professionals (HCPs), especially in low- and middle-income countries (LMICs) characterized by limited access to healthcare services due to diverse contextual and structural factors (4–7).

Interprofessional collaboration, which is essential to holistic patient care, depends mainly on mutual understanding and recognition of roles and competencies (8). An informed understanding of OT among HCPs is crucial for its integration into multidisciplinary care teams. When OT is not well understood, it can hinder teamwork, contribute to duplication of healthcare services, and result in fragmented service delivery (9). Therefore, it is crucial to raise awareness of OT and support knowledge of the profession to promote interdisciplinary collaboration and optimize patient outcomes, especially in low-resource settings.

In LMICs, many studies have shown that awareness and knowledge of OT among healthcare workers are often limited (10, 11). For instance, studies have reported persistent uncertainty regarding the scope and focus of OT practice. In a study from India, while 84.8% of HCPs acknowledged OT as an independent field, only 45.7% felt they fully understood its scope (12). Similarly, studies in Jordan and Nigeria showed that exposure to OT in clinical and educational training increased awareness of OT, but many participants still had only a partial understanding of its full scope and contributions (13, 14). Misconceptions between OT and other allied health professions, especially physiotherapy, continue to blur professional boundaries, contributing to role confusion and the underutilization of occupational services (9). Moreover, the lack of OT content in medical and allied health curricula has been cited as a barrier to knowledge acquisition in many LMICs. Enhancing health education by incorporating a comprehensive view of OT can lead to improved interdisciplinary cooperation and ultimately optimize patient outcomes (7, 15).

In Rwanda, OT is still an emerging profession that was officially introduced in 2015 with support from international collaboration initiatives (16). Today, 48 licensed occupational therapists serve a population of more than 14 million people, working in teaching hospitals, non-governmental organizations (NGOs), special education institutions, rehabilitation centers, and private clinics (17). Although the Ministry of Health has recently adopted a holistic rehabilitative approach that incorporates OT, its integration into the national health system faces persistent challenges (18, 19). Among the main barriers are a limited number of practitioners, poor visibility of the profession, lack of understanding by other HCPs, and low public awareness of OT (9). These factors restrict the potential for interdisciplinary collaboration, delay appropriate referrals, and may contribute to ineffective healthcare delivery, higher costs, and diminished satisfaction with care (20). Furthermore, Rwandan OT professionals often report a lack of recognition and misinterpretation of their roles during clinical placements and practices. Anecdotal evidence suggests that their contributions are either overlooked or undervalued, which can lead to missed opportunities for effective rehabilitation planning (21).

The scarcity of research from sub-Saharan Africa, particularly Rwanda, leaves a significant gap in understanding how OT is perceived and integrated into local healthcare systems. Most of the existing literature focuses on high-income countries, limiting its applicability to LMIC contexts where healthcare infrastructures and scopes of practice may differ substantially (13). To our knowledge, none has studied the awareness and knowledge of OT among HCPs and their associated factors in selected teaching hospitals in Rwanda. Therefore, this study aimed to fill that gap by assessing the knowledge and awareness of OT among healthcare professionals in Rwanda. Identifying the level of understanding, misconceptions, and attitudes toward OT can inform national health strategies and foster interprofessional education (IPE). This will promote better utilization of OT services. The results from this study contribute to strengthening the positioning of OT in Rwanda's growing health system, ensuring its alignment with its commitment to person-centered, rehabilitative, and community-based care.

## Methods

### Study design and area

This research employed a cross-sectional study design, which was suitable for capturing and assessing the current knowledge and awareness of OT among healthcare professionals during the period from 25 March to 6 June 2025. This study was reported in accordance with the Strengthening the Reporting of Observational Studies in Epidemiology (STROBE) guidelines for cross-sectional studies as indicated in Supplementary File 1 (22).

Rwanda is a small, landlocked country in East-Central Africa with a population of about 14 million people. It is known for its rapid post-genocide recovery and strong commitment to health system strengthening and universal health coverage. The health sector of this nation is primarily governed by the Ministry of Health, which emphasizes decentralized healthcare delivery and community-based health insurance to enhance accessibility and equity across all regions (23). The healthcare system of Rwanda operates through both public and private sectors, with the public system structured into multiple service levels. As of recent data, the public sector comprises more than 1,280 health posts, 520 health centers, and 57 district hospitals (24). Among these district hospitals, ten are designated as Level II teaching hospitals. There are also five national referral and Level I teaching hospitals. The five teaching hospitals were selected because occupational therapists were practicing at these sites. These hospitals include Rwanda Military Referral and Teaching Hospital, Ndera Neuropsychiatric Teaching Hospital, King Faisal Hospital, Byumba Level II Teaching Hospital, and Kabgayi Level II Teaching Hospital. The selected facilities have a bed capacity ranging between 100 and 500 (Supplementary File 2).

Despite notable strides in strengthening the healthcare system, Rwanda continues to experience critical workforce shortages not only in OT but also among key cadres such as nurses, psychologists, and medical doctors (MDs). The current healthcare workforce density stands at approximately one skilled professional per 1,000 population, falling significantly short of the World Health Organization's recommended threshold of four per 1,000. This disparity is particularly acute in rural and underserved areas, posing a major obstacle to achieving equitable access and universal health coverage (25). As of December 2023, the national registry recorded about 28,000 licensed HCPs, including 14,816 nurses, 2,137 midwives, 6,831 allied health professionals, 1,302 pharmacists, 102 dental surgeons, 1,303 general practitioners, and 694 specialist doctors (26, 27). However, in stark contrast, only 48 occupational therapists were actively practicing nationwide by 2025. However, occupational therapists were not found in health facilities located in rural areas. This shortage underscores a substantial deficiency in habilitation/rehabilitation services. The pipeline for training new HCPs remains narrow, with only 10–13 OT graduates entering the workplace annually. This shortage may limit the availability of habilitation/rehabilitation services and may be associated with limited familiarity with the scope and value of OT among other HCPs. Limited interprofessional exposure to occupational therapists during clinical practice and training may impede effective interdisciplinary collaboration and undermine the integration of OT into broader healthcare planning and delivery. Addressing this knowledge gap is essential to enhancing collaborative practice and optimizing patient outcomes in the evolving healthcare system of the country.

### Study population and participants

The study population included healthcare professionals working in specified teaching hospitals. These include medical doctors (general practitioners and specialists), clinical or general nurses, mental health nurses, midwives, clinical psychologists, psychiatrists, anesthesia practitioners, biomedical laboratory scientists, dental therapy practitioners, medical imaging practitioners, nutritionists, dietitians, ophthalmic clinical officers (OCOs), optometrists, speech–language therapists, physiotherapists (PTs), and prosthetics and orthotics technicians. Researchers targeted professionals directly involved in patient care to gain insights into their knowledge and awareness of occupational therapy. Inclusion criteria required participants to be (1) aged 18 years or older, (2) actively practicing in one of the five selected teaching hospitals, (3) involved in direct clinical care, (4) holding at least an A1-level professional qualification, (5) having professional experience of at least 3 months, and (6) able to understand and respond to the questionnaire in English. Exclusion criteria included (1) healthcare trainees such as students and interns, (2) administrative staff without direct clinical roles, (3) individuals working in healthcare facilities outside the selected hospitals, (4) occupational therapists to prevent potential bias, and (5) those unable or unwilling to participate due to language limitations, time constraints, lack of interest, or other reasons preventing informed consent or voluntary engagement.

### Study sampling and sample size

A convenience sampling method was employed to recruit participants for this study. This approach enabled the inclusion of all eligible healthcare professionals who were available and met the predefined inclusion criteria until the targeted sample size was achieved. Given the study's implementation across five different teaching hospitals, the research team collaborated with occupational therapists working in those institutions to facilitate contact with the respective human resources officers, who provided information on the eligible population. The total number of healthcare professionals across the selected hospitals was 1,176. The sample size was determined using Yamane's formula (28) for a finite population: *n* = N/[1 + N(e^2^)], where *n* is the sample size, N is the population size (1,176), and e is the margin of error (0.05). Applying this formula yielded a sample size of approximately 298 participants: *n* = 1176/[1 + 1,176(0.05^2^)] = 1,176/3.94 ≈ 298. This sample size was considered sufficient to ensure reliable estimates of healthcare professionals’ knowledge and awareness of occupational therapy within the selected teaching hospitals.

### Data collection and materials

The first author (DI) and the second author (EN) carried out data collection in close collaboration with the rest of the research team. Before fieldwork, all researchers received training in research ethics and the use of data collection instruments. The process was supported by the hospital administrations and occupational therapists assigned at each site, who facilitated coordination within their respective facilities. Furthermore, researchers visited the selected hospitals and, with the assistance of on-site occupational therapists, distributed questionnaires directly to healthcare professionals across various departments, including outpatient departments, internal medicine, surgery, physiotherapy, and inpatient wards. Hospitals were purposefully selected based on the presence of at least one occupational therapist, as not all hospitals in Rwanda have occupational therapy services. Eligible professionals who were available and expressed interest in participating were provided with a printed questionnaire and an informed consent form. Prior to data collection, the study objectives were briefly explained, and participants were assured of voluntary participation, anonymity, and the right to withdraw at any point without any repercussions. Participants had 1–4 weeks to complete the questionnaire privately to maintain confidentiality. Completed questionnaires were retrieved later for analysis.

### Study instruments

The Knowledge and Attitude Toward Occupational Therapy Practice Tool―a standardized questionnaire originally developed to assess the knowledge and awareness of OT healthcare professionals―was used to collect data in this study (12). This measure consisted of three parts (demographic data and professional information, including age, sex, academic qualifications, clinical experience, service provision history, working department, and facility characteristics such as hospital size; section I: awareness and knowledge and section II: advocacy). In this research, awareness of OT was defined as healthcare professionals’ prior recognition of occupational therapy as a distinct healthcare profession and was assessed by determining whether participants had previously heard of occupational therapy. Awareness of OT reflected basic familiarity with the profession but did not necessarily imply an accurate understanding of its scope of practice (29, 30). In addition, knowledge of OT was defined as healthcare professionals’ factual understanding of occupational therapy, including its roles, scope of practice, areas of intervention, target populations, and service settings. Perception referred to healthcare professionals’ beliefs, opinions, and attitudes regarding the value and contribution of occupational therapy within healthcare and interprofessional practice (12).

This tool underwent a three-phase validation process. First, occupational therapy experts assessed face validity, ensuring that the items were aligned with the intended construct, and reviewed the questionnaire. Next, a pilot test involving five healthcare professionals evaluated clarity and comprehensibility, leading to minor refinements. Finally, the revised questionnaire was administered to a larger group of 50 participants to assess internal consistency. Cronbach's alpha was calculated, yielding a value of 0.78, indicating good reliability and coherence across items.

### Data management and analysis

Collected data were initially entered into Microsoft Excel for coding and cleaning. Coding involved assigning numerical or categorical values to responses to facilitate statistical analysis. Cleaning procedures addressed missing values, entry errors, and inconsistencies. The cleaned dataset was imported into the IBM Statistical Package for Social Sciences (IBM SPSS) version 25 for statistical analysis. Descriptive statistics were used to summarize demographic characteristics and response patterns. Awareness of OT was analyzed as a binary outcome based on participants’ self-reported prior recognition of occupational therapy (29, 30). The researchers calculated the level of knowledge of OT using four questions from section I of the questionnaire: Q3, Q8, Q9, and Q10. These questions assessed the knowledge of OT among HCPs regarding the difference between OT and other professions, OT areas of practice, the types of clients who require OT, and the settings in which OT services are provided. The scores for these questions were totaled, with a maximum possible score of 33. Because no standardized or psychometrically validated cut-off scores currently exist for the Knowledge and Attitude Toward Occupational Therapy Practice Tool, knowledge scores were categorized for analytical purposes using thresholds informed by previous studies employing similar questionnaire-based approaches (13, 31, 32). Participants scoring 1–16 were classified as having poor knowledge of OT, whereas those scoring 17–33 were classified as having good knowledge of OT. Similarly, perception scores were categorized into positive (good) and negative (poor) perception using thresholds informed by previous literature (13, 29), as no validated cut-off values have been established for this instrument.

Bivariate analyses using chi-square tests were performed to examine associations between characteristics of HCPs and their levels of knowledge and awareness of OT. Variables that were statistically significant at *p* < 0.05 in the bivariate analyses were subsequently included in the corresponding multivariable logistic regression models. Specifically, variables selected from the bivariate analysis of awareness were entered simultaneously using the enter method or forced entry (33, 34) into a multivariable logistic regression model to identify factors independently associated with awareness of OT. Similarly, variables that were significant in the bivariate analysis of knowledge were entered simultaneously using the enter method into a separate multivariable logistic regression model to identify factors independently associated with knowledge of OT. Adjusted odds ratios (aORs) with 95% confidence intervals (CIs) were reported, and statistical significance at *p* < 0.05 was considered.

## Results

### Sociodemographic characteristics of the participants

Of the 298 healthcare professionals who received the questionnaires, 262 returned them, corresponding to a response rate of 87.9%. After excluding 61 questionnaires that did not meet the eligibility or completeness criteria, 201 questionnaires were included in the final analysis. The excluded questionnaires comprised 10 participants who declined participation after receiving the questionnaire, 17 questionnaires that were returned completely blank, 23 completed by students and interns rather than eligible professionals, 4 submitted by administrative personnel, and 7 that were only partially completed. Commonly cited reasons for non-completion included time constraints, high workload, limited interest, perceived lack of relevance, and the absence of incentives. Although the number of study participants (*n* = 201) was lower than the initially calculated sample size (*n* = 298), a *post hoc* power analysis indicated that the study retained an estimated statistical power ranging between 75% and 85% at a 5% significance level. This suggests an acceptable level of power, demonstrating that the achieved sample size was sufficient to detect moderate effect sizes observed in the study.

Among the 201 participants, 99 (49.3%) were male, and 102 (50.7%) were female. The largest age group was 31–40 years (*n* = 74, 36.8%), followed by 21–30 years (*n* = 69, 34.3%). These age categories reflect a moderately varied but predominantly young workforce. Regarding professional backgrounds, the most represented groups were mental health nurses (*n* = 40, 19.9%) and general nurses (*n* = 39, 19.4%). In terms of professional experience, 40.8% of participants had between 0 and 5 years of practice, indicating that a significant portion of the workforce was in the early stages of their careers. Most participants (*n* = 174, 87%) reported no previous professional practice outside Rwanda, while 13% had some form of international exposure. In addition, more than half of the participants (*n* = 113, 56%) were involved in habilitation/rehabilitation services (Table 1).

**Table 1:** Sociodemographic and health-related characteristics (*n* = 201).

Variables	Frequency	Percentage
Gender		
Male	99	49.3
Female	102	50.7
Age		
21–30	69	34.3
31–40	74	36.8
41–50	39	19.4
50+	19	9.5
Profession		
Mental health nurse	40	19.9
Nurse	39	19.4
MD	31	15.4
PT	26	12.9
OCO	9	4.5
Others	56	27.9
Years of professional experience		
0–5	82	40.8
6–10	52	25.9
11–15	25	12.5
16–20	21	10.4
20+	21	10.4
Previous professional practice outside Rwanda		
No	174	87
Yes	27	13
HCPs of habilitation/rehabilitation services		
Yes	113	56
No	88	44

MD, medical doctor; HCP, healthcare professional; PT, physiotherapist; OCO, ophthalmic clinical officers.

### Awareness of OT

Overall, 151 participants (75.1%) reported being aware of OT as a profession, whereas 50 (24.9%) reported no awareness. Among 201 HCPs surveyed, 140 (69.7%) had experience working with an occupational therapist. Among the 140 participants with awareness, 70 (50%) reported referring clients to OT whenever the case warranted, 23 (16.4%) when the client requested a referral, 14 (10.0%) often, and 33 (23.6%) seldom. OT was most commonly identified through university curricula (*n* = 82, 40.8%) and work practice (*n* = 68, 33.8%). Regarding perceived knowledge of OT, 83 participants (41.3%) agreed or strongly agreed that they felt knowledgeable about OT, whereas 68 (33.8%) disagreed or strongly disagreed, and 50 (24.9%) neither agreed nor disagreed. Regarding overall knowledge, 104 participants (51.7%) demonstrated poor knowledge, while 97 (48.3%) demonstrated good knowledge (Table 2).

**Table 2:** Awareness of occupational therapy among healthcare professionals (*n* = 201).

Variables	Frequency	Percentage
Aware of OT as a profession		
No	50	24.9
Yes	151	75.1
HCP experienced working with OT		
No	61	30.3
Yes	140	69.7
Frequency of referral to OT among HCPs who had experience working with an OT (*n* = 140)		
Whenever the case warrants	70	50.0
When the client requests such a referral	23	16.4
Often	14	10.0
Seldom	33	23.6
How HCP feel knowledgeable about OT		
Strongly disagree	16	8.0
Disagree	52	25.9
Neither agree nor disagree	50	24.9
Agree	64	31.8
Strongly agree	19	9.5
How HCP became aware of OT		
University curriculum	82	40.8
Work practice	68	33.8
Patient	5	2.5
Book/literature	10	5.0
Internet	7	3.5
Colleague	29	14.4
Level of knowledge of OT		
Poor knowledge	104	51.7
Good knowledge	97	48.3

HCPs, healthcare professionals; OT, occupational therapy.

### HCP response on the importance of OT in the healthcare team

The findings from this research demonstrated no significant associations between gender and awareness of OT (*χ*^2^ = 0.09, *p* = 0.682); however, awareness was comparable between males (75.8%) and females (74.5%). Awareness of OT did not differ significantly by age group (*χ*^2^ = 0.9, *p* = 0.0837). Although awareness ranged from 70.3% among participants aged 41–50 years to 81.3% among those aged ≥50 years, the differences were not statistically significant. In contrast, awareness differed significantly by profession (*χ*^2^ = 19.5, *p* = 0.002), with higher awareness among physiotherapists (88.5%), nurses (87.2%), and mental health nurses (87.5%) than medical doctors (54.8%), OCOs (66.7%), and other professionals (64.3%). In addition, awareness did not differ significantly according to years of clinical practice (*χ*^2^ = 3.1, *p* = 0.541) or experience of practicing in another country (*χ*^2^ = 0.7, *p* = 0.411). Awareness ranged from 64.0% among participants with 11–15 years of clinical practice to 81.0% among those with ≥20 years of practice. Awareness was 74.1% among those who had not practiced abroad and 81.5% among those who had. Similarly, the provision of habilitation/rehabilitation services was significantly associated with awareness of OT (*χ*^2^ = 15.9, *p* < 0.001), with higher awareness among participants who provided such services (85.8%) than among those who did not (61.4%). Awareness was also associated with perceptions of OT (*χ*^2^ = 17.5, *p* < 0.001) Table 3).

**Table 3:** Distribution of awareness of OT by sociodemographic and health-related characteristics.

Variables	Unaware (*n* = 50)	Aware (*n* = 151)	Chi-square	*p*-value
	N (%)	N (%)		
Gender				
Male	24 (24.2)	75 (75.8)	0.09	0.682
Female	26 (25.5)	76 (74.5)		
Age				
21–30	17 (23.6)	55 (76.4)	0.9	0.0837
31–40	19 (25)	57 (75)		
41–50	11 (29.7)	26 (70.3)		
50+	3 (18.8)	13 (81.3)		
Professions				
PT	3 (11.5)	23 (88.5)	19.5	0.002
MD	14 (45.2)	17 (54.8)		
Nurse	5 (12.8)	34 (87.2)		
Mental health nurse	5 (12.5)	35 (87.5)		
OCO	3 (33.3)	6 (66.7)		
Others	20 (35.7)	36 (64.3)		
Years of professional experience				
0–5	22 (26.8)	60 (73.2)	3.1	0.541
6–10	10 (19.2)	42 (80.8)		
11–15	9 (36)	16 (64)		
16–20	5 (23.8)	16 (76.2)		
20+	4 (19)	17 (81)		
Previous professional practice outside Rwanda				
No	45 (25.9)	129 (74.1)	0.7	0.411
Yes	5 (18.5)	22 (81.5)		
HCPs of habilitation/rehabilitation services				
No	34 (38.6)	54 (61.4)	15.9	<0.001
Yes	16 (14.2)	97 (85.8)		
Perception of HCP toward OT				
Good perception	17 (14.3)	102 (85.7)	17.5	<0.001
Poor perception	33 (40.2)	49 (59.8)		

HCP, healthcare provider; OT, occupational therapy; HCPs, healthcare providers; PT, physiotherapy; MD, medical doctor; OCO, ophthalmic clinical officers; N, frequency; %, percentage.

### Association between knowledge of OT and sociodemographic and health-related factors

Our results showed that knowledge did not differ significantly by gender (*χ*^2^ = 0.03, *p* = 0.838), with good knowledge observed among 46.5% of males and 50% of females. They also indicated no statistically significant association between age and HCPs’ level of knowledge about OT (*χ*^2^ = 4.7, *p* = 0.193). The proportion with good knowledge was 51.4% among those aged 21–30 years, 46.1% among those aged 31–40 years, 37.8% among those aged 41–50 years, and 68.8% among those aged ≥50 years. In contrast, knowledge differed significantly by profession (*χ*^2^ = 23.4, *p* < 0.001). Good knowledge was reported among 77.8% of OCOs, 76.9% of PTs, 56.4% of nurses, 52.5% of mental health nurses, 32.1% of those in other professions, and 29.0% of MDs. Knowledge was not significantly associated with years of professional experience (*p* = 0.883), with good knowledge ranging from 42.3% among those with 6–10 years of experience to 52.4% among those with more than 20 years.

HCPs who had practiced in another country had a significantly higher proportion of good knowledge than those who had not (77.8% vs. 43.7%; *χ*^2^ = 10.9, *p* < 0.001). Provision of habilitation/rehabilitation services was significantly associated with knowledge (*χ*^2^ = 44.6, *p* < 0.001), with good knowledge reported among 69.0% of HCPs who provided rehabilitation services compared with 21.6% of those who did not. Moreover, awareness of OT was also significantly associated with knowledge (*χ*^2^ = 27.7, *p* < 0.001), with good knowledge reported among 58.9% of HCPs who were aware of OT compared with 16.0% of those who were not. Working with an occupational therapist was significantly associated with knowledge (*χ*^2^ = 10.3, *p* < 0.001), with 55.7% of HCPs who worked with an occupational therapist having good knowledge. Perceptions of OT were also significantly associated with knowledge (*χ*^2^ = 58.3, *p* < 0.001), with good knowledge observed among 70.6% of HCPs with a good perception of OT compared with 15.9% of those with a poor perception (Table 4).

**Table 4:** Association between HCP level of knowledge and demographic characteristics.

Variables	Level of knowledge			
	Good	Poor	Chi-square	*P*-value
	N (%)	N (%)		
Gender				
Male	46 (46.5)	53 (53.5)	0.03	0.838
Female	51 (50)	51 (50)		
Age				
21–30	37 (53.6)	32 (46.4)	4.7	0.193
31–40	34 (45.9)	40 (54.1)		
41–50	15 (38.5)	24 (61.5)		
50+	13 (68.4)	6 (31.6)		
Profession				
PT	20 (76.9)	6 (23.1)	23.4	<0.001
MD	9 (29)	22 (71)		
Nurse	22 (56.4)	17 (43.6)		
Mental health nurse	21 (52.5)	19 (47.5)		
OCO	7 (77.8)	2 (22.2)		
Others	18 (32.1)	38 (67.8)		
Years of professional experience				
0–5	42 (51.2)	40 (48.8)	1.2	0.883
6–10	22 (42.3)	30 (57.7)		
11–15	12 (48)	13 (52)		
16–20	10 (47.6)	11 (52.4)		
20+	11 (52.4)	10 (47.6)		
Previous professional practice outside Rwanda				
No	76 (43.7)	98 (56.3)	10.9	0.001
Yes	21 (77.8)	6 (22.2)		
HCPs habilitation/rehabilitation services				
No	19 (21.6)	69 (78.4)	44.6	<0.001
Yes	78 (69)	35 (31)		
HCPs who are aware of OT as a profession				
No	8 (16)	42 (84)	27.7	<0.001
Yes	89 (58.9)	62 (41.1)		
Working with an occupational therapist				
No	19 (31.1)	42 (68.9)	10.3	0.001
Yes	78 (55.7)	62 (44.3)		
Perceptions of OT among other healthcare team members				
Poor perception	13 (15.9)	69 (84.1)	58.3	<0.001
Good perception	84 (70.6)	35 (29.4)		

OCO, ophthalmic clinical officer; N, frequency; %, percentage.

### HCP knowledge of different aspects of OT

While some respondents rated the importance of OT as very low (1), others considered it indispensable (10). Out of the 201 respondents, 82 participants (40.8%) scored below 6 (ratings 1–5), indicating negative perceptions of the importance of occupational therapy in the healthcare team (Supplementary File 3). In contrast, 119 participants (59.2%) rated OT with scores from 6 to 10, suggesting a predominantly positive perception of OT's role within a healthcare team. The results showed that most participants distinguished OT from other HCPs by its focus on improving occupational performance (*n* = 137, 33.7%) and independence in activities of daily living (ADLs) (*n* = 127, 31.3%). Participants most often identified persons with developmental disabilities (*n* = 165, 82.5%) and those with orthopedic or neurological dysfunctions (*n* = 133, 66.5%) as clients who need OT, while fewer recognized individuals with rare disorders (*n* = 33, 16.5%). Lastly, participants reported hospitals (*n* = 194, 97%) and rehabilitation centers (*n* = 167, 83.5%) as settings where occupational therapists can provide services (Supplementary File 4).

### Professional opinion on the need for OT in their workplace

Overall, 144 participants (71.6%) agreed or strongly agreed that OT plays a significant role in their workplace. Similarly, 167 participants (83.1%) agreed or strongly agreed that greater emphasis should be placed on the role of OT in their workplace. The most frequently reported reasons for the need for OT were increased functional outcomes (67.4%), better prognosis (64.8%), and greater independence in daily living (64.8%) (Table 5).

**Table 5:** Opinions of healthcare professionals on the significance and need for greater emphasis on OT in their workplace.

Variables	Frequency	Percentage (%)
Participants’ opinions on whether OT plays a significant role in their workplace		
Strongly disagree	5	2.5
Disagree	14	7.0
Neither agree nor disagree	38	18.9
Agree	39	19.4
Strongly agree	105	52.2
Participants’ views on the need for greater emphasis on the role of OT in their workplace		
Disagree	9	4.5
Neither agree nor disagree	25	12.4
Agree	49	24.4
Strongly agree	118	58.7
Participants’ opinions on why OT is needed in their workplaces		
Due to a better prognosis	125	24.2
For prevention of further ailments	81	15.7
For a patient-centric approach	55	10.7
For patients to have an independent living approach	125	24.2
For increased functional outcomes.	130	25.2

Participants could select more than one response. Percentages are based on the total number of responses.

### Multivariable logistic regression for the factors associated with awareness of OT

Multivariable logistic regression for the factors associated with awareness of OT indicated that physiotherapists [aOR = 6.3, 95% CI (1.6–25.5), *p* = 0.010], nurses [aOR = 5.6, 95% CI (1.7–18.1), *p* = 0.004], and mental health nurses [aOR = 5.8, 95% CI (1.8–18.7), *p* = 0.003] had a higher likelihood of being aware of OT than medical doctors. In contrast, ophthalmic clinical officers and other HCPs did not differ significantly from medical doctors in their awareness of OT. Furthermore, professionals involved in habilitation/rehabilitation services had greater odds of awareness of OT [aOR = 3.8, 95% CI (1.9–7.5), *p* < 0.001] than those not providing such services. The source of knowledge of OT was also associated with awareness of OT, where our results showed that those who reported learning from patients had lower odds of awareness of OT [aOR = 0.2, 95% CI (0.1–0.5), *p* = 0.008] than those who learned through the university curriculum. Similarly, those who cited the internet [aOR = 0.1, 95% CI (0.0–0.7), *p* = 0.017] or colleagues [aOR = 0.4, 95% CI (0.2–0.7), *p* = 0.006] had lower odds of higher awareness of OT than those who learned through university training. In addition, positive perceptions of OT were significantly associated with greater awareness of OT. Participants with positive perceptions had a higher likelihood of awareness of OT than those with negative perceptions [aOR = 4.1; 95% CI (2.1–8.0), *p* < 0.001] (Table 6).

**Table 6:** Multivariable logistic regression for the factors associated with awareness of OT.

Variable	aOR	*p*-value	95% confidence intervals	
			Lower	Upper
Profession				
Medical doctor	1			
Physiotherapist	6.3	0.010	1.6	25.5
Nurse	5.6	0.004	1.7	18.1
Mental health nurse	5.8	0.003	1.8	18.7
Ophthalmic clinical officer	1.7	0.530	0.4	7.8
Others	1.5	0.388	0.6	3.6
Provide habilitation/rehabilitation services				
No	1			
Yes	3.8	<0.001	1.9	7.5
How HCP became aware of OT				
University curriculum	1			
Work practice	0.7	0.333	0.3	1.5
Patient	0.2	0.008	0.1	0.5
Book/literature	0.8	0.738	0.1	4.0
Internet	0.1	0.017	0.0	0.7
Colleagues	0.3	0.006	0.2	0.7
Perception toward OT				
Poor perception	1			
Good perception	4.1	<0.001	2.1	8.0

OT, occupational therapy; aOR, adjusted odds ratio.

Multivariable logistic regression models indicated that physiotherapists had significantly higher odds of good knowledge of OT [aOR = 8.2, 95% CI (2.5–27.0), *p* = 0.001] than medical doctors. Similarly, ophthalmic clinical officers [aOR = 8.6, 95% CI (1.5–49.4), *p* = 0.016], general nurses [aOR = 3.2, 95% CI (1.2–8.6), *p* = 0.024], and mental health nurses [aOR = 2.7, 95% CI (1.1–7.3), *p* = 0.045] had higher odds of good knowledge of OT than medical doctors. Conversely, other HCPs did not show significant associations with knowledge of OT. Furthermore, participants who had practiced in another country besides Rwanda had higher odds of good knowledge of OT than those who had only practiced in Rwanda [aOR = 4.5, 95% CI (1.7–11.7), *p* = 0.002]. Similarly, healthcare professionals providing habilitation or rehabilitation services had higher odds of good knowledge of OT [aOR = 8.1, 95% CI (4.2–15.4), *p* < 0.001] than non-providers of such healthcare services. Being aware of OT as a profession was significantly associated with good knowledge of OT. Participants who were aware of OT had higher odds of demonstrating good knowledge of OT [aOR = 7.5, 95% CI (3.3–17.2), *p* < 0.001] than those who were not aware of the profession. In addition, working with occupational therapists was associated with a greater likelihood of having good knowledge of OT [aOR = 2.8, 95% CI (1.5–5.3), *p* = 0.002] than not working or practicing with them. Finally, participants with a positive perception of OT as an important member of the healthcare team had higher odds of good knowledge than those with a poor perception [aOR = 12.7, 95% CI (6.3–25.9), *p* < 0.001] (Table 7).

**Table 7:** Multivariable logistic regression for the factors associated with the knowledge of OT.

Variables	aOR	*p*-value	95% confidence intervals	
			Lower	Upper
Current profession				
Medical doctors	1			
Physiotherapist	8.2	<0.001	2.5	27.0
Nurse	3.2	0.024	1.2	8.6
Mental health nurse	2.7	0.045	1.1	7.3
OCO	8.6	0.016	1.5	49.4
Others	1.2	0.764	0.5	3.0
Practiced in any other country than Rwanda				
No	1			
Yes	4.5	0.002	1.74	11.7
Provide habilitation/rehabilitation services				
No	1			
Yes	8.1	<0.001	4.2	15.4
Awareness of OT as a profession				
No	1			
Yes	7.5	<0.001	3.3	17.2
Work and practice with an occupational therapist				
No		1		
Yes	2.8	0.002	1.5	5.3
Importance of occupational therapist in healthcare team				
Bad perception	1			
Good perception	12.7	<0.001	6.3	25.9

OT, occupational therapy; aOR, adjusted odds ratio.

## Discussion

Our study aimed to examine the level of knowledge and awareness of OT among HCPs and their associated factors. Overall, 75.1% of participants recognized OT as an independent profession. This rate is lower than reported in Nigeria and South Africa (13, 35). Our findings revealed that physiotherapists had higher odds of being aware of OT than medical doctors. This may reflect their frequent collaboration with occupational therapists in rehabilitation teams, where both professions work closely to support patient recovery (36). Similarly, mental health professionals had higher odds of being aware of OT than medical doctors. This could be related to the long-standing integration of OT in mental health services. In these situations, the role of OT is distinct, focusing on meaningful activities and psychosocial interventions central to recovery, such as art-based therapy and music therapy. This distinct contribution contrasts with physical rehabilitation, where overlapping roles sometimes obscure the visibility of OT (37). Furthermore, general nurses showed a greater likelihood of being aware than medical doctors, which is consistent with their routine collaboration with OTs in rehabilitation teams, where shared patient care may be associated with a greater understanding of professional roles (36).

Professionals actively involved in habilitation and rehabilitation showed higher odds of awareness of OT than those not involved in such services, suggesting a positive association between habilitation/rehabilitation practice and awareness of OT. These results corroborate previous studies (30, 31). Importantly, the source of knowledge of OT was also associated with awareness of OT. HCPs who reported learning about OT through patients, colleagues, or the internet demonstrated lower levels of awareness of OT than those taught through the university curriculum. These findings highlight an association between formal educational exposure and greater awareness of OT. In addition, positive perceptions were associated with awareness of OT compared to those with negative perceptions. This could be because some HCPs began to understand the role of OTs in their respective workplaces and had opportunities to collaborate with OT professionals to provide adequate healthcare services, although this association warrants further investigation. These findings disagree with those reported in a study conducted in Virginia, USA, where HCPs acknowledged the importance of OT in a healthcare team (38).

Despite relatively high awareness, most participants demonstrated poor knowledge of OT. Similar patterns have been documented in other sub-Saharan African (SSA) countries regarding OT (39, 40). This indicates persistent knowledge gaps that may hinder collaboration, referral practices, and optimal utilization of OT services. One major explanation for this concern could be a lack of OT content in professional training and insufficient exposure during training or professional practice.

Regarding associated factors of knowledge of OT, professional category was an important factor in knowledge of OT. Being physiotherapists, ophthalmic clinical officers, nurses, and mental health nurses was associated with higher odds good knowledge of OT more than being medical doctors. This likely reflects closer collaboration between habilitation/rehabilitation professionals and OTs, which may be associated with greater understanding. These findings align with previous studies reporting that physicians have limited awareness and knowledge of OT (41). Conversely, other HCPs did not show significant associations with knowledge of OT. Furthermore, the provision of habilitation and rehabilitation services was another strongly associated factor, as professionals in these fields were significantly more likely to possess good knowledge of OT compared to non-providers. These results are consistent with other research showing that habilitation/ rehabilitation professionals tend to have greater familiarity with OT due to shared training environments and workplace integration (42). Medical doctors, however, displayed predominantly poor knowledge of OT. This may be explained by limited curricular exposure to OT during medical training and fewer opportunities to engage with OTs in practice. Similarly, age and years of experience were not associated with knowledge of OT, differing from findings in other contexts where experience enhanced interprofessional collaboration (38). This discrepancy may reflect the relative novelty of OT in Rwanda.

Being aware of OT as a profession was associated with higher odds of good knowledge of OT than those who were unaware. Working with occupational therapists was associated with a greater likelihood of having good knowledge of OT than not working or practicing with them. Respondents with a good perception of OT as an important member of the healthcare team had higher odds of good knowledge of OT than their counterparts with a poor perception. These results are supported by previous studies that indicated that healthcare professionals perceive OT as an important profession (9). While other studies have documented the age of HCPs and their years of practice as important determinants of knowledge (14, 42), our study found no statistically significant association between age, years of practice, and knowledge of OT.

Moreover, those with previous professional practice outside Rwanda were more knowledgeable about OT than those without such experiences. This discrepancy may reflect greater integration and visibility of OT in high-income health systems, where OT is well established and recognized across disciplines (43, 44). By contrast, in many LMICs, including Rwanda, OT remains poorly defined and under-promoted; however, it has made recent progress due to current efforts by various nations (13, 14). This underscores the urgent need for internal advocacy, education, and capacity building to advance the professional identity of OT and interprofessional collaboration. Lastly, HCPs who provide habilitation or rehabilitation services had higher odds of having good knowledge of OT than those who do not provide habilitation services. These results align with findings indicating that members of the rehabilitation team, such as speech therapists, audiologists, and physical therapists (PTs), have a greater understanding of the role of occupational therapy (OT) (9, 45). This may be explained by the fact that OT is part of the rehabilitation professions; professionals in these fields often study together in universities and work in the same department in hospitals. Finally, HCPs who viewed OT as an important profession were more likely to have good knowledge of OT. Similarly, those who worked directly with OTs demonstrated good knowledge of OT, which underscores the role of interprofessional education and workplace collaboration. These findings align with previous studies (36, 42).

### Study strengths and limitations

This study had several strengths. To our knowledge, this is the first study to assess HCPs’ knowledge and awareness of OT in Rwanda. This contribution is particularly important given that occupational therapy is a new profession in the country and remains limited in availability and representation within health professional education and research. The small number of practicing occupational therapists, the limited number of students entering the profession, and the emerging research capacity in occupational therapy further highlight the need for empirical evidence to support the profession's development and integration within the Rwandan healthcare system. Second, the study was conducted in five selected teaching hospitals where occupational therapists are actively practicing, allowing for the assessment of the knowledge and awareness of HCPs who have been exposed to OT services.

This study had some limitations. First, the use of convenience sampling and self-administered questionnaires could introduce response bias, as participants might have overestimated or underestimated their knowledge or given socially desirable answers. Second, the cross-sectional study design allowed us to examine associations but did not allow us to make causal inferences. Third, although the final sample size did not reach the initially calculated target, a *post hoc* power analysis indicated acceptable statistical power (0.75–0.85), suggesting that the achieved sample size was sufficient to detect the observed effect sizes. However, statistical power does not establish representativeness or generalizability. Furthermore, the inclusion of only hospitals with practicing occupational therapists, together with the use of convenience sampling, may limit the generalizability of the results, as participants were selected based on accessibility rather than random sampling. Furthermore, as participants were recruited from five hospitals, the analyses did not account for potential hospital-level clustering, which may have underestimated standard errors and should be considered when interpreting the findings. In addition, some HCPs did not participate or did not complete the questionnaire because they were unavailable during the data collection period, had time constraints, heavy workloads, lacked interest, or because no incentives were provided. Moreover, as no standardized or validated cut-off values currently exist for the “Knowledge and Attitude Toward OT Practice Tool”, the categorization of “good” and “poor” knowledge and perception was based on thresholds adopted from previous studies using similar questionnaire-based approaches. These thresholds were selected to facilitate logistic regression analyses and should not be interpreted as clinically validated classifications. Finally, the absence of qualitative data limited a deeper exploration of participants’ perceptions and experiences regarding OT.

## Conclusion

Our study indicated that awareness of OT was relatively high among HCPs; however, more than half of the participants showed poor knowledge of the profession. Awareness was significantly associated with professional group, involvement in habilitation or rehabilitation services, and positive perceptions of OT. Good knowledge was associated with professional group, previous professional practice outside Rwanda, involvement in habilitation/rehabilitation services, awareness of occupational therapy, experience working with occupational therapists, and positive perceptions of the profession. The findings indicate that awareness of OT was not necessarily accompanied by adequate knowledge of its scope and role. Strengthening IPE, continuing professional development (CPD), and institutional strategies that enhance OT visibility and role clarity may help address these knowledge gaps and support more effective interprofessional collaboration within Rwanda’s healthcare system. Future scholars should investigate these associations using larger, representative, and where appropriate, longitudinal or mixed methods designs to better understand how exposure to OT and interprofessional collaboration influence knowledge and perceptions among HCPs. Lastly, intervention-based research could assess the effectiveness of IPE and CPD initiatives in strengthening OT knowledge and role clarity within the Rwandan healthcare system.

## Data Availability

The original contributions presented in the study are included in the article/Supplementary Material, further inquiries can be directed to the corresponding author.
