# Supplementary material for: Knowledge and awareness of occupational therapy and associated factors among healthcare professionals in Rwanda: a cross-sectional study
Source: Front Med (Lausanne). 2026 Oct 20;13:1722607. doi: 10.3389/fmed.2026.1722607 (PMC13727406; doi:10.3389/fmed.2026.1722607)
Supplement: Supplementary file 1 [file Table1.docx]

**Supplementary Table 1: Distribution of healthcare professionals and an expected sample from each hospital**

| **Hospitals** | **Number** | **Percentage** | **Sample** |  |
| --- | --- | --- | --- | --- |
| RMRTH | 407 | 34.6 | 103 |  |
| NNPTH | 251 | 21.34 | 64 |  |
| KFHR | 198 | 16.8 | 50 |  |
| Byumba level II teaching hospital | 169 | 14.4 | 43 |  |
| Kabgayi level II teaching hospital | 151 | 12.8 | 38 |  |
| **Total** | **1176** | **100** | **298** |  |
| *Notes:* ***KFHR:*** *King Faisal Hospital Rwanda****, NNPTH:*** *Ndera Neuropsychiatric Teaching Hospital,* ***RMRTH:*** *Rwanda Military Referral and Teaching Hospital* | | | | |
