# Supplementary material for: Knowledge and awareness of occupational therapy and associated factors among healthcare professionals in Rwanda: a cross-sectional study
Source: Front Med (Lausanne). 2026 Oct 20;13:1722607. doi: 10.3389/fmed.2026.1722607 (PMC13727406; doi:10.3389/fmed.2026.1722607)
Supplement: Supplementary file 2 [file Table2.docx]

**Supplementary Table 2: Participants’ ratings of the importance of OT in the healthcare team**

| **RATING (1-10)** | **FREQUENCY** | **PERCENTAGE** |
| --- | --- | --- |
| 1 (Not important) | 5 | 2.5 |
| 2 | 12 | 6 |
| 3 | 13 | 6.5 |
| 4 | 18 | 9 |
| 5 | 34 | 16.9 |
| 6 | 19 | 9.5 |
| 7 | 20 | 10 |
| 8 | 19 | 9.5 |
| 9 | 14 | 7 |
| 10 (Indispensable) | 47 | 23.4 |
| **Total** | 201 | 100 |
