# Supplementary material for: Knowledge and awareness of occupational therapy and associated factors among healthcare professionals in Rwanda: a cross-sectional study
Source: Front Med (Lausanne). 2026 Oct 20;13:1722607. doi: 10.3389/fmed.2026.1722607 (PMC13727406; doi:10.3389/fmed.2026.1722607)
Supplement: Supplementary file 3 [file Table3.docx]

**Supplementary Table 3: HCP knowledge of different aspects of OT**

| **Variables** | **Knowledge** | |
| --- | --- | --- |
|  | **Frequency** | **(%)** |
| **How OT is different from other professions** | | |
| OT focuses on providing independence in ADL | 127 | 31.3 |
| OT focuses on improving or restoring occupational performance | 137 | 33.7 |
| OT focuses on functional ambulation rather than gait training | 59 | 14.5 |
| OT enhances functional performance rather than a specific movement | 83 | 20.4 |
| **Participants’ knowledge of the OT’s scope of practice** | | |
| Activities of daily living | 167 | 12.5 |
| Sensory integration | 93 | 6.9 |
| Hand therapy | 137 | 10.2 |
| Ergonomics | 83 | 6.2 |
| Splinting | 69 | 5.2 |
| Cardiopulmonary/respiratory training | 42 | 3.1 |
| Home safety assessment, evaluation, and modification | 71 | 5.3 |
| Psychosocial intervention | 76 | 5.7 |
| Community based rehabilitation | 108 | 8.1 |
| Acute care interventions | 50 | 3.7 |
| NICU early interventions | 39 | 2.9 |
| Geriatric care | 89 | 6.6 |
| Assistive technology | 83 | 6.2 |
| Handwriting skills | 108 | 8.1 |
| Driving rehabilitation | 72 | 5.4 |
| Disability certification | 52 | 3.9 |
| **Participants’ knowledge of clients who need OT** | | |
| Persons with developmental disabilities | 165 | 24.6 |
| Persons with functional impairments due to orthopedic/neurological dysfunctions | 133 | 19.8 |
| Persons with psychiatric disorders | 110 | 16.4 |
| Elders with dementia | 102 | 15.2 |
| Individuals who experience difficulty with self-care, work, education, play, and leisure activities. | 128 | 19.1 |
| Rare disorders | 33 | 4.9 |
| **Participants’ knowledge on settings where OTs practice** | | |
| Hospitals | 194 | 22.9 |
| Rehabilitation centers | 167 | 19.7 |
| Outpatient clinics | 110 | 13 |
| Nongovernmental organizations for people with disabilities | 101 | 11.9 |
| Home based occupational therapy | 112 | 13.2 |
| Schools/special schools | 121 | 14.3 |
| Teleconsultation | 41 | 4.8 |
| *Notes:* ***OT****: Occupational Therapists; ADL: Activity of daily living;* ***NICU****:* Neonatal Intensive Care Units | | |
